# Protein Misfolding and ER Stress in Huntington's Disease

**DOI:** 10.3389/fmolb.2019.00020

**Published:** 2019-04-03

**Authors:** Talya Shacham, Neeraj Sharma, Gerardo Z. Lederkremer

**Affiliations:** ^1^Sagol School of Neuroscience, Tel Aviv University, Tel Aviv, Israel; ^2^George Wise Faculty of Life Sciences, School of Molecular Cell Biology and Biotechnology, Tel Aviv University, Tel Aviv, Israel

**Keywords:** Huntington's disease, neurodegeneration, protein misfolding, protein aggregation, ER stress, unfolded protein response

## Abstract

Increasing evidence in recent years indicates that protein misfolding and aggregation, leading to ER stress, are central factors of pathogenicity in neurodegenerative diseases. This is particularly true in Huntington's disease (HD), where in contrast with other disorders, the cause is monogenic. Mutant huntingtin interferes with many cellular processes, but the fact that modulation of ER stress and of the unfolded response pathways reduces the toxicity, places these mechanisms at the core and gives hope for potential therapeutic approaches. There is currently no effective treatment for HD and it has a fatal outcome a few years after the start of symptoms of cognitive and motor impairment. Here we will discuss recent findings that shed light on the mechanisms of protein misfolding and aggregation that give origin to ER stress in neurodegenerative diseases, focusing on Huntington's disease, on the cellular response and on how to use this knowledge for possible therapeutic strategies.

## Protein Misfolding and Aggregation

### Protein Synthesis, Folding and Misfolding

The acquisition of a correct three-dimensional native structure is a crucial step during the biosynthesis of a functional cellular protein (Vendruscolo et al., 2003). Protein folding is an intrinsic property of a polypeptide chain, which is strongly influenced by the cellular environment and is under regulation of other partner proteins in oligomeric assemblies and of folding catalysts and molecular chaperones (Hartl and Hayer-Hartl, 2002; Ellis and Minton, 2006). During co-translational folding, exposed regions of partially folded proteins are at risk of interaction with other cellular molecules (Hartl and Hayer-Hartl, 2002; Balchin et al., 2016; Chiti and Dobson, 2017). Therefore, short non-native intermediate forms develop to protect the regions which are susceptible to aggregation (Mogk et al., 2018). Proteins undergo a stochastic search for more stable conformations with lowest free energy by a trial and error process (Dinner et al., 2000; Vendruscolo et al., 2003; Chiti and Dobson, 2017). In general, the marginally stable native state of a folded protein and the crowded cellular environment are responsible for the error proneness of protein folding, despite being always under inspection of the protein quality control system, the ubiquitin-proteasome complex and molecular chaperones (Vendruscolo et al., 2003; Labbadia and Morimoto, 2015; Miller et al., 2015; Balchin et al., 2016). Failure of any of these protective checkpoints can initiate a protein misfolding process, which can generate protein oligomers or larger aggregates. The failure can be caused, among others, by high temperature, low pH, oxidative stress, abnormal presence of metal ions, mutations, transcriptional, translational or posttranslational errors, and aging (Sekijima et al., 2005; Chu et al., 2007; Chiti and Dobson, 2009; Koga et al., 2011; Brehme et al., 2014; Mckinnon and Tabrizi, 2014; Labbadia and Morimoto, 2015; Martinez-Lopez et al., 2015; Balchin et al., 2016).

### Protein Aggregation and Its Effects in Disease

A protein can form amorphous aggregates or amyloid fibrils with well-ordered straight fibrillar structure, composed of protofilaments (Chiti and Dobson, 2017; Hartl, 2017). Conditions that support high net protein charge favor the formation of fibrillar aggregates.

Protein aggregation can follow several pathways that are not mutually exclusive. Native protein monomers may have a natural tendency to reversibly associate and to form small oligomers, which can occasionally associate into large aggregates. Alternatively, the native monomer may have a very low tendency to reversibly associate but it can undergo a conformational change resulting in an aggregation-prone misfolded form. This misfolded conformation may also arise from mutations. In a surface-induced aggregation mechanism, aggregation begins with the binding of a native monomer to the surface of the container *in vitro* or to intracellular membranes in cells. These interactions cause conformational changes in the monomer which increase its tendency to aggregate (Philo and Arakawa, 2009). For any of these mechanisms, it is widely accepted that progression into large aggregates involves a nucleation and seeding mechanism. Independently of the lower or higher tendencies to form small oligomers, once an aggregate of sufficient size (critical nucleus) forms, then it grows exponentially. Two kinetic phases are observed: a lag phase generating a seed and an elongation phase where visible particulates appear (Soto and Pritzkow, 2018). Seeding activity was recently found at early stages, before appearance of visible aggregates, in pre-symptomatic HD model mice (Ast et al., 2018).

**Figure 1:**
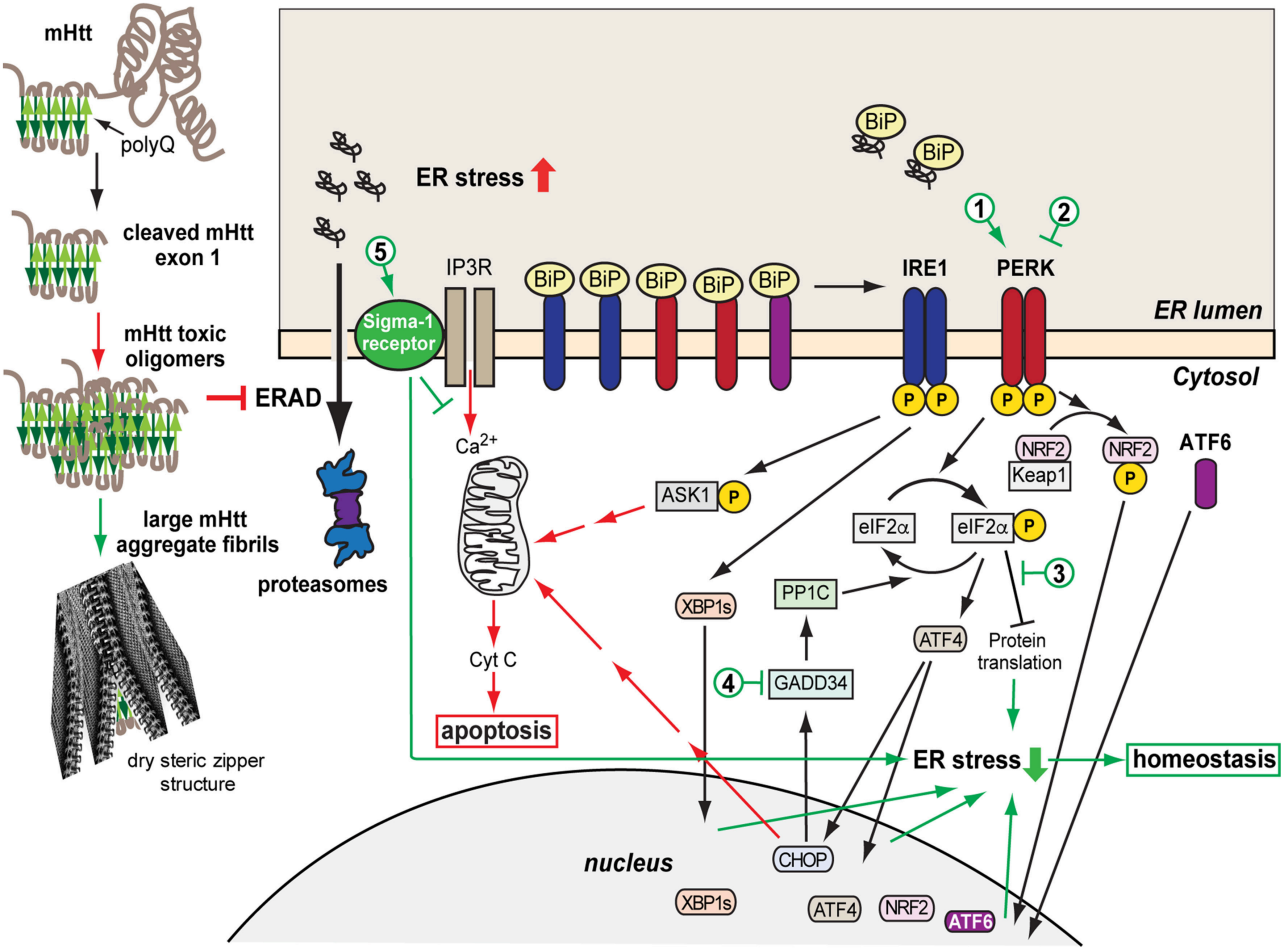
Model of mHtt aggregation, generation of ER stress and the consequent UPR protective and later pro-apoptotic responses. A misfolded mHtt monomer, with the aggregation prone polyQ domain indicated (beta sheets in green) is cleaved and associates with other monomers to form toxic oligomers, which among other effects cause sequestration and depletion of ERAD factors such as p97, inhibiting ERAD. mHtt oligomers can associate into larger aggregate fibrils with dry steric zipper structure that shield the toxicity of mHtt. However, the transient presence of toxic mHtt oligomers, inhibiting ERAD, causes accumulation of unfolded secretory proteins and ER stress, activating the UPR sensors IRE1, PERK, and ATF6, starting an initial protective or adaptive phase of the UPR. This includes upregulation and translocation to the nucleus of transcription factors, XBP1s, ATF4, NRF2, ATF6, which induce expression of chaperones, ERAD machinery, anti-oxidative response components. Concurrently, eIF2α phosphorylation by PERK causes transient arrest in translation, reducing the ER load. If the ER stress remains unresolved, the pro-apoptotic stage of the UPR is initiated, causing upregulation of ASK1-P and CHOP and Ca^2+^ exit from the ER, inducing the intrinsic apoptotic pathway with the mitochondrial release of cytochrome C. Sigma-1 receptor is upregulated and modulates Ca^2+^ release, with a protective effect. Cytotoxic pathways are indicated in red and cell protective ones in green. The numbers indicate possible points of therapeutic intervention, with activation (1) or inhibition (2) of PERK, inhibition of the downstream effects of eIF2α-P (3), inhibition of eIF2α-P dephosphorylation (4), and Sigma-1 receptor activation (5).

Genetic, biochemical, mouse model studies, and neuropathological evidence have shown that in neurodegenerative diseases misfolded proteins form aggregates, in most cases amyloid fibrils, which may be deposited intracellularly or extracellularly. In diseases, such as Alzheimer's disease (AD), Parkinson's disease (PD), Amyotrophic Lateral Sclerosis (ALS), and HD, protein aggregates vary, but all forms have a similar intermolecular beta-sheet–rich structure, both in small oligomers and in large fibrillar aggregates. The implicated polypeptides are different but they can form similar structures of amyloid fibrils (Eisenberg and Jucker, 2012; Chiti and Dobson, 2017; Hartl, 2017). The aggregation process triggers cellular dysfunction, loss of synaptic connections and is responsible for brain damage or disease (Soto, 2003; Ross and Poirier, 2004; Goedert, 2015; Soto and Pritzkow, 2018). Initial studies hypothesized that the large insoluble aggregates lead to cell death, and therefore prevention of aggregate formation might be a strategy to help reduce the toxicity (Bucciantini et al., 2002). However, there is increasing evidence that in general, smaller soluble toxic misfolded oligomers are the main causative agent for neurodegeneration (Jarrett et al., 1993; Morris et al., 2009; Leitman et al., 2013; Chiti and Dobson, 2017; Hartl, 2017; Moily et al., 2017). The 3D structure of the oligomeric species appears to determine their toxic character (Hoffner and Djian, 2015; Smith, 2018). The insoluble aggregates could then be a result of a strategy of the cells to reduce the toxicity by shielding the toxic species in these large structures called dry steric zippers (Eisenberg and Jucker, 2012) (Figure 1).

### Protein Aggregation in HD and Other Neurodegenerative Diseases

HD is a result of mutation in the gene encoding for the huntingtin protein (Htt), the expansion of CAG repeats that encode for a polyglutamine (polyQ) stretch, which is pathogenic when it contains more than about 35 glutamines (Zoghbi and Orr, 2000; Sakahira et al., 2002). A similar phenomenon occurs in other polyQ diseases, such as spinocerebellar ataxias (Shao and Diamond, 2007). In HD, the mutation results in mutant Htt (mHtt) misfolding and aggregation. The polyQ stretch is on exon 1, which is cleaved off, with the resulting N-terminal fragment enough to cause aggregation. A recent report shows also the presence of aberrantly spliced mHtt forms containing only exon 1 in samples from HD patients (Neueder et al., 2017). The 17-residue-long N-terminus preceding the polyQ stretch interacts with intracellular membranes and has a strong influence on mHtt aggregation (Pandey et al., 2017). Ubiquitination at the N-terminus appears to be an important determinant for mHtt degradation (Wang et al., 2017). In a recent study, a mutation in the N17 N-terminal region prevented the formation of large aggregates but not oligomers in a Drosophila HD model and led to an increase in toxicity. This suggested neurotoxicity of the oligomers and perhaps protection by the large aggregates (Branco-Santos et al., 2017). This follows a growing number of reports linking mHtt oligomers and not the final large aggregates to cytotoxicity (Schaffar et al., 2004; Takahashi et al., 2008; Lajoie and Snapp, 2010; Leitman et al., 2013). In fact, no connection was found between large mHtt aggregates and neuronal death in HD patients (Kuemmerle et al., 1999). In cellular HD models, the transition from oligomers to large aggregates correlates with a reduction in ER stress (Leitman et al., 2013) and in apoptosis (Ramdzan et al., 2017). However, the formation of mHtt inclusions still affects to some degree cellular function (Ramdzan et al., 2017). As mentioned above, the toxicity of soluble oligomers is not unique to HD. No correlation was found between large insoluble aggregates and memory loss in an AD mouse model (Lesné et al., 2008) and AD patients do not necessarily present brain aggregates (Petersen et al., 2013). Targeting oligomeric forms of Aβ instead of large aggregates provides better results in the reduction of AD toxicity (Sengupta et al., 2016).

Misfolded proteins trigger cellular responses, such as the heat shock response (Kakkar et al., 2014; Kampinga and Bergink, 2016) and the ER or mitochondrial unfolded protein responses (UPR). These responses attempt to increase the capacity to unfold and refold the misfolded proteins through upregulation of molecular chaperones and to improve the ability to degrade the misfolded proteins by upregulation of the proteasome machinery. These responses are cell autonomous but a non-cell autonomous regulation has been described, especially in studies in *Caenorhabditis elegans* (Prahlad and Morimoto, 2011; Volovik et al., 2014).

Failure of the UPR or of the heat-shock response are found to be associated with several pathologic conditions such as AD, PD, HD, prion diseases, Type II diabetes, and ALS (Douglas and Dillin, 2010; Lee et al., 2011; Hipp et al., 2014; Labbadia and Morimoto, 2015; Chiti and Dobson, 2017; Hartl, 2017; Shamsi et al., 2017). Progressive decline in the efficiency of these pathways with age has been linked to the late age of onset of these diseases (Taylor and Dillin, 2011). The UPR and its failure in disease are discussed in the following chapters.

## ER Stress and Huntington's Disease

### ER Stress and the UPR

ER stress develops in many neurodegenerative diseases such as AD, PD, ALS, and HD (Ogen-Shtern et al., 2016; Xiang et al., 2016; Remondelli and Renna, 2017) and there is increasing evidence that it is a main factor in the degeneration of the cells (Hoozemans et al., 2005; Vidal et al., 2011; Stutzbach et al., 2013; Heman-Ackah et al., 2017). Many of these diseases are a result of mutation in specific genes (e.g., Htt, SOD1) leading to the accumulation of a misfolded protein. As mentioned above, misfolded proteins have a propensity to interact and aggregate in the cell, giving rise to toxic species that generate ER stress and compromise cell function. One of the ways by which ER stress is generated in this process, in many cases by cytosolic proteins such as mHtt, is by interference of toxic oligomers with ER-associated degradation (ERAD) components (Figure 1). This has been observed in HD (Duennwald and Lindquist, 2008; Leitman et al., 2013) and AD models (Abisambra et al., 2013; Soejima et al., 2013; Fonseca et al., 2014). ER stress triggers activation of the UPR, with the aim of removing or refolding the damaged proteins (Hetz and Papa, 2017). The UPR is activated through three main transmembrane proteins acting as UPR sensors: IRE1, PERK, and ATF6 (Figure 1). Inositol-requiring protein 1α (IRE1) is a protein kinase associated in its inactive form with the chaperone Binding immunoglobulin Protein (BiP). Upon accumulation of unfolded proteins, IRE1 dissociates from BiP/GRP78 and becomes active by autophosphorylation. When active, IRE1 splices the mRNA encoding for the transcription factor XBP-1, which is then translated to the active form XBP-1s. XBP-1s translocates into the nucleus, inducing transcription of BiP and other chaperones and target genes related to ERAD regulation (Chen and Brandizzi, 2013). A second sensor associated with BiP is PKR-like ER-localized eIF2α kinase (PERK). Activation of PERK by autophosphorylation, after dissociation from BiP, results in phosphorylation of eukaryotic initiation factor 2α (eIF2α), leading to inhibition of protein translation. Paradoxically, translation of the transcription factor ATF4 increases upon phosphorylation of eIF2α (Ron, 2002; McQuiston and Diehl, 2017), leading to expression of many genes, among them Growth arrest and DNA damage-inducible gene 34 (GADD34, also called PPP1R15A), a regulatory subunit of protein phosphatase PP1, which dephosphorylates eIF2α, resulting in recovery from the PERK-mediated translational block (Novoa et al., 2001). Besides eIF2α, PERK phosphorylates nuclear erythroid 2 p45-related factor 2 (NRF2), causing dissociation of the cytosolic NRF2-Keap1 complex and leading to release of NRF2 to the nucleus where it promotes transcription of RNAs encoding components of the antioxidant response (Cullinan et al., 2003). The third UPR sensor is activating transcription factor 6 (ATF6) which is regulated by BiP as well. Upon BiP dissociation, ATF6 translocates from the ER to the Golgi, where it is cleaved by proteases that detach ATF6 from the Golgi membrane. The released soluble cytosolic domain of ATF6 is a transcription factor that enters the nucleus and activates expression of UPR target genes such as BiP and XBP1 (Yoshida et al., 2001; Shen et al., 2002; Hetz, 2012). ER stress activates the three UPR branches, leading to inhibition of protein translation, increase in chaperone production and enhanced degradation. However, a chronic activation of the UPR leads to a fatal outcome. In case of failure to restore protein homeostasis, the UPR initiates an apoptotic pathway leading to cell death. IRE1 mediates activation of tumor necrosis factor receptor associated factor 2 (TRAF2), activating apoptotic factors such as ASK1. A long-term phosphorylated status of eIF2α induces CHOP/GADD153, a transcription factor that activates expression of pro-apoptotic genes (Oyadomari and Mori, 2004; Sano and Reed, 2013). PERK activation and eIF2α phosphorylation without resolution of ER stress have been particularly implicated in neurodegeneration (Ohno, 2017; Taalab et al., 2018).

### ER Stress and Cell Toxicity in HD

HD is a genetic disorder characterized by movement disorder, cognitive decline, and behavioral difficulties (Walker, 2007; Wright et al., 2017; Pandey and Rajamma, 2018). As found in post-mortem and MRI studies, HD patients suffer from neuronal cell death, initially and mostly in the striatum but also in the cortex and other areas of the brain (Reiner et al., 1988; Rosas et al., 2003). Although the involvement of protein aggregation in neurodegenerative disease is well established (Murphy, 2002; Taylor, 2002; Ross and Poirier, 2004), toxicity could arise through a number of pathways. In HD, protein aggregation in the cytoplasm interferes for example with nucleocytoplasmic transport, causing aberrant redistribution of nuclear shuttle factors to the cytosol (Woerner et al., 2016). Over-expression of molecular chaperones, such as Hsp70 and Hsp40, was shown to reduce the toxicity of mHtt aggregates in yeast and fly HD models, without preventing their formation, possibly by sheltering the exposed hydrophobic regions or by a conformational effect on the misfolded protein (Kazemi-Esfarjani and Benzer, 2000; Meriin et al., 2002). Another important consequence of mHtt aggregation is the activation of the UPR (Duennwald and Lindquist, 2008; Reijonen et al., 2008; Carnemolla et al., 2009; Leitman et al., 2013, 2014; Jiang et al., 2016). Interference with the ubiquitin-proteasome system (UPS) is important in HD models and apparently also in HD patients (Bennett et al., 2007; Ortega et al., 2007; Finkbeiner and Mitra, 2008; Hipp et al., 2012). However, some studies using cytosolic GFP-based reporters of proteasomal activity did not find a global UPS impairment (Bowman et al., 2005; Bett et al., 2009). In fact, an important interference was attributed to sequestration and depletion of p97/VCP and its cofactors Npl4 and Ufd1 by mHtt, which would not cause global UPS deficiency, but rather a crippling dysfunction of ERAD (Duennwald and Lindquist, 2008; Yang et al., 2010; Leitman et al., 2013). Homocysteine-induced endoplasmic reticulum protein (Herp), an important factor in ERAD, was recently reported to be directly involved in targeting of mHtt for degradation (Luo et al., 2018). The inhibition of ERAD leads to accumulation of unfolded proteins in the ER, ER stress, and UPR induction, which were observed in HD models in yeast and mammalian cells (Duennwald and Lindquist, 2008; Reijonen et al., 2008; Carnemolla et al., 2009; Leitman et al., 2013, 2014), in animal HD models (Carnemolla et al., 2009; Cho et al., 2009; Noh et al., 2009; Vidal et al., 2012), and in post-mortem samples from HD patients (Carnemolla et al., 2009). Other cellular factors, such as ubiquitin-specific protease-14 and ATF5, are important for reduction of ER stress, and were recently found to be sequestered and depleted during mHtt aggregation (Hyrskyluoto et al., 2014; Hernández et al., 2017). One of the consequences of ER stress is to affect mitochondrial function and exacerbate oxidative stress, a key element in mHtt cytotoxicity (reviewed in Zheng et al., 2018).

## Sigma-1 Receptor

One important ER stress-activated protein, with a cell-protective function is the Sigma-1 receptor (S1R). The S1R is an evolutionarily conserved ligand–operated molecular chaperone, especially involved in brain function, including neuromodulation and neuroplasticity (Hayashi, 2015). The S1R is expressed in a wide range of tissues, with higher levels in the central nervous system (CNS), where it is linked to diverse pathologies (Nguyen et al., 2017). The S1R is a transmembrane protein (25 KD) consisting of 223 amino acids and largely localized to a specialized ER subdomain that contacts mitochondria and is called the mitochondrion–associated ER membrane (MAM) (Hanner et al., 1996; Hayashi and Su, 2007; Hayashi et al., 2009; Fujimoto and Hayashi, 2011; Kourrich et al., 2012). The overall topology of this receptor is still unclear and there are studies that suggest a single or two transmembrane domains (Hayashi and Su, 2007; Ortega-Roldan et al., 2015; Schmidt et al., 2016; Mavylutov et al., 2017). A recent crystal structure of the S1R suggests a single transmembrane domain and oligomerization into trimeric and up to hexameric assemblies (Schmidt et al., 2016). The S1R is activated by both ER stress and agonists, which might lead to its translocation to different subcellular compartments, such as the MAM, nuclear envelope, and plasma membrane (Hayashi and Su, 2003; Su et al., 2010; Kourrich et al., 2012). The association of BiP with the S1R keeps it in an inactive conformation (Hayashi and Su, 2007; Tsai et al., 2014). Upon ER stress, accumulation of misfolded proteins in the ER causes S1R dissociation from BiP and its activation. Calcium levels and S1R agonists also modulate its activity (Hayashi and Su, 2007; Wang et al., 2012), and S1R expression is upregulated in response to PERK pathway activation (Mitsuda et al., 2011). The “chaperone” activity of S1R is through modulation of inositol 1,4,5-triphosphate receptor (IP3R) activity. IP3R is a ligand-gated calcium channel that upon activation releases calcium from the ER, especially at the MAMs (Li et al., 2009; Gilady et al., 2010). Therefore, S1R modulation of IP3R activity regulates calcium transfer to mitochondria (Hayashi and Su, 2007) promoting ATP generation and attenuating the mitochondrial apoptotic pathway (Hayashi and Su, 2007; Bernard-Marissal et al., 2015; Hayashi, 2015). Studies have also demonstrated a suppressor effect of S1R activity on ROS and oxidative damage (Meunier and Hayashi, 2010; Pal et al., 2012; Wang et al., 2012).

A decreased level of S1R or its activity is found associated with neurodegenerative diseases (Nguyen et al., 2015). A S1R mutation was linked to a familial form of ALS (Al-Saif et al., 2011). The mutation impairs MAMs and affects calcium signaling (Bernard-Marissal et al., 2015). Several *in vivo* and *in vitro* studies have shown evidence of S1R as a target for treating neuropsychiatric as well as neurodegenerative disorders (Katnik et al., 2006; Meunier et al., 2006; Vagnerova et al., 2006; Mancuso et al., 2012; Behensky et al., 2013; Hyrskyluoto et al., 2013; Francardo et al., 2014; Nguyen et al., 2014). S1R was reported to have a protective effect in HeLa cells expressing mHtt, increasing proteasome activity and mHtt degradation (Miki et al., 2015). In the next section we will discuss recent studies on HD models, which reported a beneficial effect of S1R modulation.

## Therapeutic Approaches for HD

There is currently no cure or effective therapy for HD. There are two main recent experimental approaches to try to develop a therapy (reviewed in Huang et al., 2016; Wright et al., 2017; Caron et al., 2018; Saavedra et al., 2018). One involves gene therapy, which includes knockdown of mHtt (Aguiar et al., 2017; Datson et al., 2017; Southwell et al., 2018) and expression of miRNAs (Miniarikova et al., 2017; Evers et al., 2018) (Table 1). Htt is necessary in embryonic development but it might be possible to eliminate it in the adult brain, (reviewed in Liu and Zeitlin, 2017). Allele-specific CRISPR/Cas9-mediated gene editing has been recently reported in adult HD140Q-knockin mice (Yang et al., 2017). CRISPR/Cas9 can also be used to specifically remove the CAG repeats (Dabrowska et al., 2018). A main set-back of gene therapy approaches is attaining efficient delivery, but there is huge progress in this direction in recent years. Therapy involving fetal cell transplantation has also been attempted (Bachoud-Lévi et al., 2006; Mazzocchi-Jones et al., 2009; Schackel et al., 2013; Precious et al., 2017). The other approach focuses on blocking the cellular toxicity of mHtt. This includes many strategies, for example inhibiting mHtt cleavage by caspase 6 (Aharony et al., 2015), targeting or modulating heat shock proteins (Kampinga and Bergink, 2016; Scior et al., 2018), neurotrophic factors such as CNTF (Emerich et al., 1997), HDACs (Suelves et al., 2017), proteasome activity (Jeon et al., 2016), and mitochondrial oxidative phosphorylation (Ruetenik et al., 2016). Compounds that promote daf-16/FOXO function and in turn stimulate insulin/IGF1 signaling and extend longevity (Hesp et al., 2015), such as resveratrol, metformin and some steroids, have shown beneficial effects in *C. elegans* (Farina et al., 2017) and mouse HD models (Arnoux et al., 2018). Metformin also appeared protective in a statistical analysis of HD patients participating in the Enroll-HD database (Hervas et al., 2017).

**Table 1:** Therapeutic approaches for HD.

**Approach**	**Strategy**	**Target**	**Model/drug**	**References**
Reduction of ER stress and/or mHtt toxicity	Activation of protein quality control	Trimeric Hsp70, Hsp110, Hsp40 chaperone	HD patient-derived neural cells and *C. elegans* HD model	Scior et al., 2018
		PDI	N171-82Q HD model mouse/LOC14	Zhou et al., 2018
		P97/VCP	HD cell model	Leitman et al., 2013
		P97-mHtt interaction	HD mouse- and patient-derived cells/peptide	Guo et al., 2016
		PERK	HdhQ111 cell model/A4	Leitman et al., 2014
		Protein misfolding	R6/2 mice/chemical chaperones	Ferrante et al., 2003 Keene et al., 2002
		Proteasome activity	YAC128 HD mice/PA28-gamma	Jeon et al., 2016
		Ca^2+^ balance	YAC128 HD mice/EVP4593	Wu et al., 2018
	Activation of mitochondrial function	OXPHOS	Yeast and Drosophila polyQ models	Ruetenik et al., 2016
	Activation of autophagy	XBP1, ATF4	XBP1- or ATF4-deficient mice, YAC128 mice, HdhQ111 knock-in mice	Vidal et al., 2012
		Lysosomal function	HEK293 HD cell model/genistein	Pierzynowska et al., 2018
		ENC1	SH-SY5Y cells, embryonic HD mice striatum	Lee et al., 2016
	Activation of Sigma-1 receptor	Sigma-1 receptor	Neuronal PC6.3 cell HD model/Pre084, pridopidine	Hyrskyluoto et al., 2013.
			R6/2 and Yac128 HD mouse models/Pre084, pridopidine	Garcia-Miralles et al., 2017 Kusko et al., 2018 Squitieri et al., 2015
			Cells from YAC128 HD mice/3-PPP, pridopidine	Bol'shakova et al., 2017 Ryskamp et al., 2017
			Clinical trials/pridopidine	Waters et al., 2018
	Other approaches	Caspase 6—mHtt cleavage	BACHD mice/peptide	Aharony et al., 2015
		Excitotoxicity	Sprague Dawley rats injected with quinolinic acid/CNTF	Emerich et al., 1997
		HDAC3	HdhQ111 knock-in mice/RGFP966	Suelves et al., 2017
		daf-16/FOXO	*C. elegans* HD model 128Q/ steroids, resveratrol	Farina et al., 2017
			Hdh150 knock-in mice/metformin	Arnoux et al., 2018
			Statistical analysis of Enroll-HD patients/metformin	Hervas et al., 2017
Gene therapy	CRISPR/Cas9- knockout	mHtt gene	Adult HD140Q-knockin mice	Yang et al., 2017
	CRISPR/Cas9- CAG repeats editing	mHtt gene	HD patient-derived fibroblasts	Dabrowska et al., 2018
	mHtt knock down	CAG repeat in mHtt mRNA (antisense)	R6/2 mice model	Datson et al., 2017 Southwell et al., 2018
	miRNA expression	mHtt mRNA (AAV5-miHTT)	HD (tgHD) minipig model	Evers et al., 2018
			Sprague Dawley rats injected with a LV expressing a chimeric mHtt fragment	Miniarikova et al., 2017
Stem cell therapy	Fetal cell transplantion	Fetal striatal cells	C57/BL6 mice with striatal lesion	Mazzocchi-Jones et al., 2009
			Sprague Dawley rats injected with quinolinic acid	Schackel et al., 2013
			HD patients	Bachoud-Lévi et al., 2006

Reduction of ER stress is protective in HD, and can be accomplished for example with the use of chemical chaperones (Keene et al., 2002; Ferrante et al., 2003). As mentioned above, p97/VCP depletion is an important factor in HD pathogenicity, through inhibition of ERAD and development of ER stress (Leitman et al., 2013). It was recently reported that VCP interacts with mHtt on mitochondria, enhancing mitophagy and cell death; a peptide was developed to inhibit the interaction (Guo et al., 2016). There is a cross-talk between ER stress and autophagy and for example, XBP1 deficiency leads to an enhancement of autophagy and overall improvement in HD model mice (Vidal et al., 2012). Induction of autophagy by genistein was protective in a cellular HD model (Pierzynowska et al., 2018). An ER stress upregulated protein, ectodermal-neural cortex 1 (ENC1), was recently shown to inhibit autophagy in an HD cellular model, and its knockdown increased autophagy and cell survival, suggesting it as a possible target for therapy (Lee et al., 2016). mHtt-induced ER stress also increases expression of protein disulfide isomerase (PDI) (Duennwald and Lindquist, 2008), a small molecule PDI modulator caused improved motor function and survival in the N171-82Q HD model mouse (Zhou et al., 2018). ER Ca^2+^ depletion causes protein misfolding in the ER and ER stress. Recent studies targeted Ca^2+^ balance, showing beneficial effects, with a small molecule activator of SERCA in a rat model of PD (Dahl, 2016) and with inhibition of TRPC1-Dependent Store-Operated Calcium Entry in an HD mouse model (Wu et al., 2018).

### Targeting the UPR Pathways

One strategy with encouraging results so far for several neurodegenerative diseases is to target the UPR pathways (Shenkman et al., 2015; Xiang et al., 2016; Pérez-Arancibia et al., 2017). PERK, one of the three UPR sensors, is an interesting and promising target (recently reviewed in Halliday et al., 2017a; McQuiston and Diehl, 2017; Ohno, 2017; Hughes and Mallucci, 2018; Taalab et al., 2018). A very low activity of PERK-mediated eIF2α phosphorylation in striatal neurons in culture and in the mouse brain striatum was found connected to the higher mHtt toxicity in this region (Leitman et al., 2014). As explained above, initial activation of PERK is beneficial, through phosphorylation of eIF2α, resulting in transient inhibition of protein synthesis and by activation of NRF2 and its anti-oxidant effects. However, chronic ER stress leads to upregulation of CHOP through the PERK pathway, which triggers a series of events ending in apoptosis. Therefore, inhibition of PERK can be beneficial, by reducing the activation of the apoptotic pathway, but this will preclude the initial protective role of PERK (Figure 1). Conversely, activating or prolonging eIF2α phosphorylation can improve the initial protective stage but might lead to apoptosis if it is not controlled. Both strategies have been attempted, with positive results, but also with some negative reports. The PERK inhibitor GSK2606414 was protective in prion (Moreno et al., 2013), frontotemporal dementia (Radford et al., 2015) AD (Yang et al., 2016) and PD (Mercado et al., 2018) mouse models but secondary side effects of pancreatic toxicity appeared as well, as high levels of insulin production in the pancreas require regulation by a functional PERK pathway. Compounds that inhibit the pathway downstream of eIF2α-P, and restore protein synthesis, were developed and showed protective effects in neurodegeneration caused by prion infection and in frontotemporal dementia, without the secondary toxicity (ISRIB, Trazodone) (Sidrauski et al., 2013; Halliday et al., 2015, 2017b). In several tauopathies, PERK variants with reduced activity are a genetic risk factor. Expression of these variant alleles in iPSC-derived neurons, or inhibition of PERK, showed high vulnerability to ER stress in these cells (Yuan et al., 2018), suggesting that an approach involving PERK pathway activation would be beneficial. In a strategy to increase eIF2α-P, GADD34 inhibitors (salubrinal, guanabenz, sephin 1) were tested, showing beneficial effects in mouse models of prion disease (Das et al., 2015), PD (Sun et al., 2018), and ALS (Tsaytler et al., 2011), but accelerated disease progression in another ALS study (Vieira et al., 2015). Another approach is to directly activate PERK. A PERK activator, CCT020312, was developed (Stockwell et al., 2012) and recently tested in mouse and cellular tauopathy models, showing beneficial effects and no toxicity (Bruch et al., 2017). PERK modulation has only been reported in HD cellular models (Leitman et al., 2014), but our own unpublished studies in mouse HD models, using a PERK activator that we have developed, showed significant protection. A combinatorial drug approach, targeting different UPR pathways, or concomitantly modulating other mechanisms has not been tried and may be advantageous.

### Approaches Involving the Sigma-1 Receptor

As mentioned above, S1R, activated by ER stress, could be a promising target for ameliorating symptoms in HD (Nguyen et al., 2015; Bol'shakova et al., 2017). The treatment of an mHtt expressing neuronal cell line (PC6.3) with the S1R agonist PRE-084 had a neuroprotective effect by restoring the S1R deficit, upregulating antioxidant activity, and decreasing ROS levels via NF-kB signaling (Hyrskyluoto et al., 2013). This agonist and also 3-PPP showed a neuroprotective effect by increasing the density in neuronal cultures from HD model mice (Bol'shakova et al., 2017). Another S1R agonist, pridopidine (also known as a dopamine stabilizer) showed a neuroprotective effect in the R6/2 and Yac128 HD mouse models, improving motor performance and survival (Squitieri et al., 2015; Garcia-Miralles et al., 2017; Kusko et al., 2018), mainly through normalization of calcium homeostasis (Ryskamp et al., 2017). Our own unpublished results in HD cellular models suggest that the effect of pridopidine through S1R activation is by modulation of ER stress, affecting especially the PERK pathway. A series of clinical trials have found some encouraging results for pridopidine therapy in HD (Squitieri and De Yebenes, 2015; Waters et al., 2018).

## Concluding Remarks

Recent evidence increases our understanding of the consequences of protein misfolding and aggregation in HD on the generation of ER stress and cytotoxicity. Given the similarities in the origin of ER stress in multifactorial neurodegenerative diseases such as AD, the findings on HD have important wider implications. It is becoming more clear that the toxicity originates mainly from soluble oligomeric assemblies rather than the large protein inclusions that develop with time. Therefore, there is less interest in the development of inhibitors of aggregation, a strategy that has failed so far. Late onset has been linked to failure with age of protein homeostasis and the consequent development of uncontrolled ER stress. HD therapy is not yet imminent, but recent work points the way, with modulation of the UPR, especially of the PERK pathway. The question of whether to inhibit or activate this pathway should probably be rephrased, as the solution might be different for each disease and the aim should be to restore PERK pathway activity to an optimal middle point. Other therapeutic strategies could be the targeting of UPR-regulated downstream factors, such as the promising recent reports of modulation of the S1R in HD.

## Author Contributions

All authors listed have made a substantial, direct and intellectual contribution to the work, and approved it for publication.

### Conflict of Interest Statement

The authors declare that the research was conducted in the absence of any commercial or financial relationships that could be construed as a potential conflict of interest.
